# Current Understanding of the Pathogenesis of Dengue Virus Infection

**DOI:** 10.1007/s00284-020-02284-w

**Published:** 2020-11-24

**Authors:** Puneet Bhatt, Sasidharan Pillai Sabeena, Muralidhar Varma, Govindakarnavar Arunkumar

**Affiliations:** 1 Manipal Institute of Virology, Manipal Academy of Higher Education, Manipal, Karnataka 576104 India; 2 Dept of Infectious Diseases, Kasturba Medical College, Manipal Academy of Higher Education, Manipal, Karnataka 576101 India; 3 Present Address: WHO Country Office, Kathmandu, Nepal

## Abstract

The pathogenesis of dengue virus infection is attributed to complex interplay between virus, host genes and host immune response. Host factors such as antibody-dependent enhancement (ADE), memory cross-reactive T cells, anti-DENV NS1 antibodies, autoimmunity as well as genetic factors are major determinants of disease susceptibility. NS1 protein and anti-DENV NS1 antibodies were believed to be responsible for pathogenesis of severe dengue. The cytokine response of cross-reactive CD4+ T cells might be altered by the sequential infection with different DENV serotypes, leading to further elevation of pro-inflammatory cytokines contributing a detrimental immune response. Fcγ receptor-mediated antibody-dependent enhancement (ADE) results in release of cytokines from immune cells leading to vascular endothelial cell dysfunction and increased vascular permeability. Genomic variation of dengue virus and subgenomic flavivirus RNA (sfRNA) suppressing host immune response are viral determinants of disease severity. Dengue infection can lead to the generation of autoantibodies against DENV NS1antigen, DENV prM, and E proteins, which can cross-react with several self-antigens such as plasminogen, integrin, and platelet cells. Apart from viral factors, several host genetic factors and gene polymorphisms also have a role to play in pathogenesis of DENV infection. This review article highlights the various factors responsible for the pathogenesis of dengue and also highlights the recent advances in the field related to biomarkers which can be used in future for predicting severe disease outcome.

## Introduction

Dengue infection is a major public health problem and has been reported from the Americas, Africa, Southeast Asia, Europe, Western Pacific, and Eastern Mediterranean regions. This arboviral disease is found to be endemic in more than 100 countries and around 96 million infected individuals are symptomatic with varying levels of severity [1, 2]. Dengue is one of the leading causes of significant morbidity and economic burden in different regions across the world including Southeast Asia and the Indian subcontinent [3].

Dengue is a mosquito-borne *Flavivirus* infection, primarily transmitted by *Aedes aegypti* followed by *Aedes albopictus* mosquito and other species of genus *Aedes* [1, 4]*.* There are four serotypes of dengue virus which are antigenically distinct namely DENV-1, DENV-2, DENV-3, and DENV-4 [5]. A fifth serotype (DENV-5) has been detected using isolation and genetic sequence analysis in Sarawak state of Malaysia in October 2013 [6].

The incubation period of dengue virus infection is 4–7 days. The disease spectrum ranges from asymptomatic infection and moderate febrile illness (dengue fever) to more serious manifestations such as dengue hemorrhagic fever (DHF) and dengue shock syndrome (DSS) [7]. The most severe clinical syndrome can manifest in the form of dengue shock syndrome (DSS), which also includes coagulation abnormalities, plasma leakage, and increased vascular fragility. The fluid loss due to increased capillary permeability leads to hypovolemic shock and multi-organ failure [8]. Every year, dengue virus infection results in approximately 20,000 deaths especially among secondary dengue cases associated with DHF/DSS [8, 9].

Till 2008, dengue was classified according to 1997 WHO classification criteria into dengue fever, dengue hemorrhagic fever (DHF), and dengue shock syndrome (DSS) [10]. The current revised WHO 2009 case classification system categorizes symptomatic cases into dengue without warning signs, dengue with warning signs, and severe dengue [11, 12].

The pathogenesis of dengue virus infection and severe dengue manifestations is very complex and not completely understood. The pathophysiological hallmark of DHF/DSS is plasma leakage and deranged hemostasis. Even after being aware of plasma leakage in dengue since the last five decades, the clear-cut mechanism of this manifestation stills remains obscure [13]. The statement that the human immune response plays a key role in the pathogenesis of the disease is favored by the fact that DENV infection displays the most severe form when the virus is being cleared by the host immune system and not with the peak viral load [14].

Various studies have been carried out across the world emphasizing the role of several factors implicated in the pathogenesis of dengue in humans. Despite a plethora of literature available on the pathogenesis of dengue fever, there are still some gaps in our knowledge, which represent a critical challenge in understanding the concepts of disease pathogenesis and severe manifestations.

The present article reviews the current concepts of the various mechanisms involved in the pathogenesis of dengue virus infection and gives a comprehensive overview of the multiple factors responsible for severe clinical manifestations of the disease. This review article also gives a brief insight into the recent advances and research in dengue pathogenesis and the role of various biomarkers as early predictors of dengue disease severity.

## Pathogenesis of Dengue

The four dengue virus serotypes (DENV1–4) have a 65–70% nucleotide sequence homology and are closely related [15]. Primary infection is defined as the initial or first infection with a certain serotype. Most of primary infections are usually asymptomatic or manifest as a mild febrile illness, although they can also cause hemorrhagic fever in some patients, especially in infants born to DENV-immune mothers. Subsequent infection with a different serotype is known as secondary dengue infection and may lead to severe clinical manifestations such as dengue hemorrhagic fever (DHF) or dengue shock syndrome (DSS) [16–18].

After an infection with a particular serotype, an individual is immune to re-infection with the same serotype. However, infection with a different serotype can occur subsequently, as the heterologous immunity is short-lived. Based on many cohort studies, the heterotypic protective immunity gradually wanes in 1 or 2 years subsequently [19].

The pathogenesis of dengue was attributed to various viral and host factors such as non-structural protein 1 (NS1) viral antigen, DENV genome variation, subgenomic RNA, antibody-dependent enhancement (ADE), memory cross-reactive T cells, anti-DENV NS1 antibodies and autoimmunity. The severe dengue manifestations in humans are mainly ascribed to the synergistic effect of all the above-mentioned factors.

**Fig. 1:**
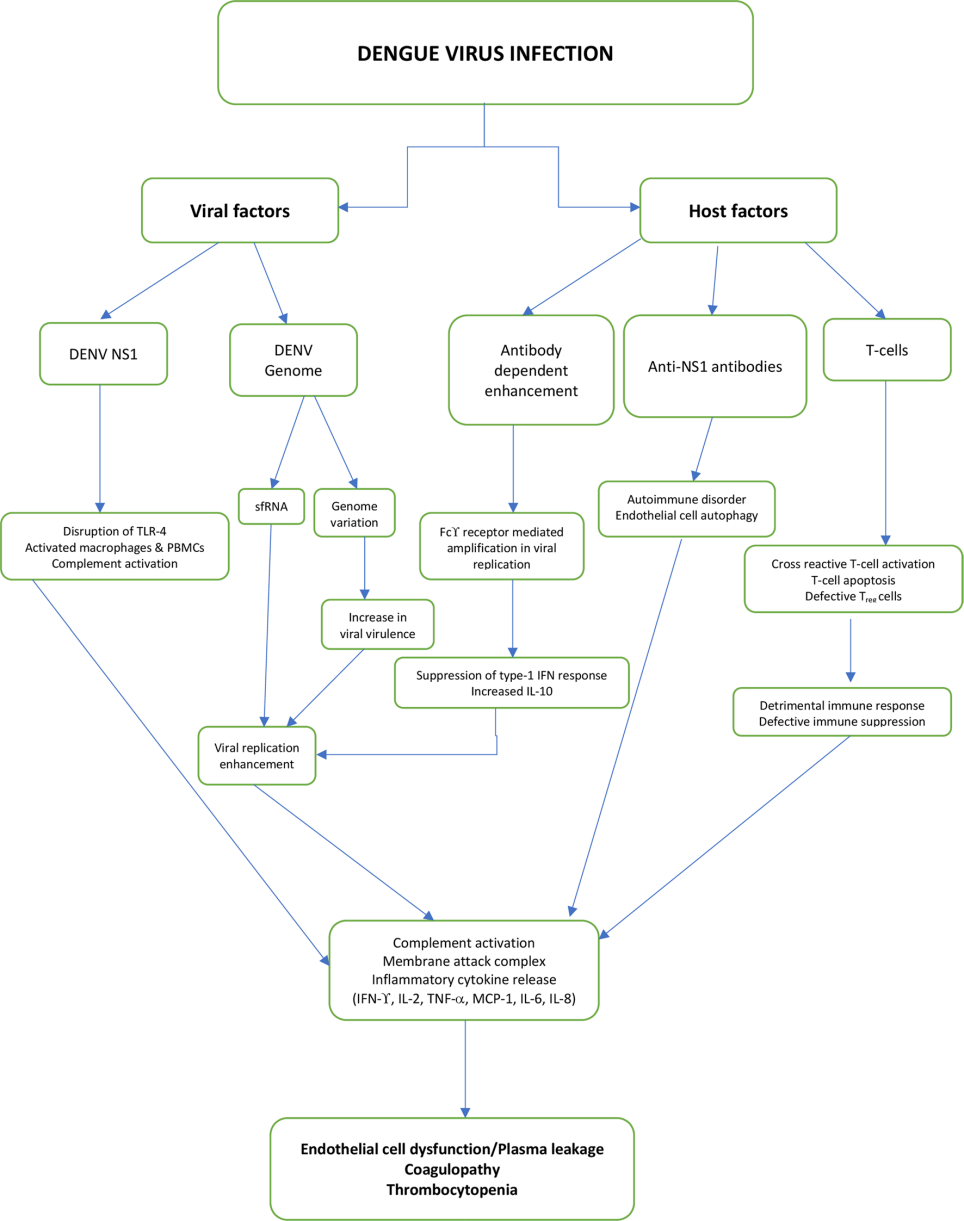
Complex interplay of viral and host factors in pathogenesis of dengue virus infection

These various factors which play a role in the pathogenesis of dengue virus infections are shown in Fig. 1. Clinical and epidemiological studies report the association of second heterotypic dengue infections as well as primary dengue infections in infants of dengue-immune mothers with dengue vascular permeability syndrome (DVPS) [20–22].

### Role of Non-structural Protein 1 (NS1) Viral Antigen

Dengue virus genome encodes for three structural proteins (C, prM (M), and E) and seven non-structural (NS) proteins (NS1, NS2a, NS2B, NS3, NS4a, NS4B, and NS5). The most important non-structural protein which has been implicated in the pathogenesis of dengue viral infections is NS1 [23]. Multiple oligomeric forms exist in humans which are found both on the cell surface (m-NS1) and as a soluble, secreted lipoparticle (s-NS1) [24]. During the acute phase of the disease, the levels of s-NS1 are particularly high, which correlates with the disease severity [25, 26]. DENV NS1 antigen was observed to be a major factor causing disruption of endothelial cell monolayer integrity as this protein has a direct action on vascular endothelium. Dengue NS1 can cause disruption of endothelial cell monolayer integrity by eliciting inflammatory cytokine production due to activation of macrophages and human peripheral blood mononuclear cells (PBMCs) through Toll-like receptor 4 (TLR 4) [8, 27]. It has been observed in mouse model experiments that the endothelial permeability was increased by DENV NS1 in a dose-dependent manner. Moreover, endothelial permeability returned to normal after administration of anti-NS1 antibodies. NS1 also induces shedding of heparan sulfate proteoglycans, which leads to the disruption of the endothelial glycocalyx layer in human pulmonary vascular endothelial cells and resulted in loss of sialic acid from cell surface. All these factors contributed to the increase in vascular permeability by direct action of NS1 antigen [28].

Meanwhile, NS1-mediated release of inflammatory cytokines from immune cells also contributes to endothelial hyperpermeability and vascular leak [27, 29]. DENV NS1 can directly trigger complement activation by the alternative pathway, targeting liver cells which leads to the stimulation of inflammatory cytokines. This subsequently leads to plasma leakage and accumulation of fluid in third space, ultimately causing dengue shock syndrome [30].

Dengue NS1 causes complement activation which is a host defense mechanism against the infection. It helps in opsonization of viral particles. The end result of complement activation is the lysis of target cells through the assembly of the membrane attack complex (MAC), which is composed of complement proteins C5b-C9. This The C5b-C9 complex can stimulate robust expression of inflammatory cytokines that are associated with DHF development. C5b-C9 complex formation which is often associated with DHF has been found to have a significant association with DENV NS1 levels. The activation of complement also generates anaphylatoxins C5a and C3a, which also play a significant role in the inflammatory process [8, 31]. Moreover, it has also been seen that the gene expression of the complement inhibitor CD59 was upregulated more strongly in peripheral blood mononuclear cells (PBMCs) from non-severe dengue patients than in DHF patients [32, 33]. Furthermore, it has also been observed the anti-DENV antibodies activated complement on the surface of endothelial cells resulting in membrane attack complex formation [33, 34].

DENV NS1-mediated activation of cathepsin L/heparanase pathway and endothelial sialidases may lead to disruption of the endothelial glycocalyx-like layer (EGL) in vitro, which was corroborated by the finding that inhibition of these enzymes is sufficient to prevent vascular leak induction by DENV2 NS1 [35]. Chen et al. reported that macrophage migration inhibitory factor (MIF) is secreted due to DENV NS1, which might induce autophagy in human endothelial cell line [8, 36].

Few studies have reported augmented DENV NS1 levels in the development of DHF/DSS. DENV NS1 antigen was also reported to be persisting for a longer duration in patients with vascular leakage, leading to the manifestations of severe dengue [37–39]. In contrast, few studies also report that DENV NS1 levels were similar in primary and secondary dengue irrespective of the severity [40].

Despite the numerous gaps in our knowledge and inadequate comprehension of the basic structure and function of DENV NS1, many studies have shown that intracellular NS1, co-localizing with dsRNA, plays an essential cofactor role in viral replication [25, 41, 42]. However, the precise nature of its cofactor function is yet to be elucidated [43]. The intracellular NS1, in its dimeric form, along with other non-structural proteins and viral RNA are targeted towards the endoplasmic reticulum (ER), which leads to the formation of a replication complex (RC) from ER-derived membrane structures called Vesicle Packets (VPs). These replication complexes play an important part in active viral replication, as the viral replication happens in these complexes [44]. In a study by Mackenzie JM et al., the subcellular localization of NS1 was investigated both by immunofluorescence and cryo-immunoelectron microscopy of infected Vero and C6/36 cells. They demonstrated that both NS1 and dsRNA probes colocalized with vesicle packets in infected Vero cells and virus-induced vacuoles in infected C6/36 cells. It is possible that NS1 may fulfill a structural function in the vesicles that comprise the replication complex, thus suggesting a possible role of NS1 protein in DENV genome replication [45].

In a study by Libraty et al. comprising only DENV-2 patients, mean plasma levels of NS1 were found to be higher in DHF patients than that of DF patients on the third day of illness and trended even higher fifth day [46]. Duyen et al. had demonstrated that the NS1 kinetics varied markedly by serotype and immune status.

A considerably higher NS1 concentration was observed in DENV-1 infection than in DENV-2 infections and a rapid decrease in NS1 concentration in secondary infection than in primary infection from the fifth day of illness was noted. This might be a result of early IgG response in secondary DENV infections leading to immune complex formation, resulting in severe clinical manifestations [47]. The reason for higher NS1 levels in DENV-1 than that in DENV-2 infections is not completely clear and it does not imply that DENV-1 infections are more severe than DENV-2 infections. These intra-serotypic differences in NS1 levels cannot be completely explained and have to be multifactorial.

In a study by Cruz-Hernandez et al., it was demonstrated that in patients with DENV-1 infections, higher NS1 levels were associated with DHF than those with DF. However, these findings were not found in patients with DENV-2 infections [48]. Meanwhile, contrasting results were demonstrated by Fox et al., who observed statistically insignificant association between NS1 and DHF [49]. The inconsistent results in these studies might be attributed to the different population, different geographical region and on the sample size.

### Anti-DENV NS1 Antibodies and Autoimmunity

Apart from NS1 protein, anti-DENV NS1 antibodies were believed to be responsible for pathogenesis of severe dengue. A major factor that is considered responsible for severe dengue pathogenesis is disorganized release of cytokines. Several cytokines and chemokines are secreted by the human endothelial cells, in response to anti-DENV NS1 antibodies.

The importance of the role played by anti-NS1 antibody mediated immune response in the development of manifestations of severe dengue cannot be undermined [8]. Lin et al. demonstrated that in addition to cell apoptosis, anti-DENV NS1 stimulation resulted in immune activation in endothelial cells. It has been suggested that anti-NS1 antibodies bind to GPI-anchored NS1 proteins on cell membranes, resulting in activation of cellular signal transduction pathways and tyrosine phosphorylation of cellular proteins. DENV NS1 antibodies, in a NF-κB-dependent manner, can stimulate the release of numerous inflammatory mediators [50]. These include MCP-1, IL-6 and IL-8. It has been observed that elevated levels of IL-6 and IL-8 correlated with clinical manifestations of DHF, and endothelial cells infected with DENV induced IL-6 and IL-8 production. In the study by Lin et al., it was shown that MCP-1 production by endothelial cells was enhanced after anti-DENV NS1 antibody stimulation. MCP-1-mediated elevation of ICAM-1 expression and facilitation of leukocyte transmigration have been suggested [51–53]. Results in this study showed that MCP-1 up-regulated ICAM-1 expression in anti-DV NS1-treated HMEC-1 cells. Endothelial cell activation followed by elevated expression of ICAM-1 may contribute to the adherence of immune cells to endothelial cells in the inflammatory responses associated with dengue disease pathogenesis.

In a study by Mehta VK et al., it was observed that serum and CSF levels of IL-6 and IL-8 were significantly higher in dengue patients with neurological manifestations as compared to controls [54]. In another study, the levels of IL-6 and IL-8 were found to be significantly higher in DHF cases as compared to that in DF cases. The levels of IL-8 seemed to be more relevant to DHF pathogenesis, as it also correlated with thrombocytopenia and raised alanine transaminase (ALT) [55]. IL-6, being a pro-inflammatory cytokine, plays a significant role in pathogenesis of severe dengue infection, along with other cytokines such as IL-1 and TNF-α. IL-8 levels were also observed to be increased in patients of DHF. In a study, more than 50% patients of DHF who died had IL-8 levels above 200 pg/ml. IL-8 may also be responsible, in part, for the intravascular coagulation seen in DHF patients [7]. These findings suggest an important role of these cytokines in increasing the severity of disease and death.

Liver damage is a vital manifestation of severe dengue disease and it is postulated that anti-DENV NS1 antibodies might also have a role to play. This can be proved by actively immunizing mice with DENV NS1 or passively administering anti-DENV NS1 antibodies and demonstrating a hepatitis-like pathogenic effect [51, 52].

In a mouse model study carried out by Lin C-F et al., it was demonstrated that anti-DENV NS1 antibodies bound to vascular endothelium in the portal and central veins of naive mouse liver. In this study, various pathological changes were seen in histological examination of the liver tissues of DENV NS1-immunized mice, which included hepatic fibrosis, fatty liver, cell infiltration, necrotic body, and vesicle formation. It was also observed that serum AST and ALT levels were much higher in mice administered anti-DENV NS1 IgG. Liver tissue sections from mice passively immunized with anti-DENV NS1 showed antibody deposition in vascular endothelium and increase of infiltrated mononuclear phagocytic cells, which may be responsible for liver injury [52]. There can be multiple causes of liver injury in cases of dengue hemorrhagic fever. Liver histopathology in fatal cases of DHF has revealed that hepatocytes and Kupffer cells may be the target cells for viral replication and an apoptotic mechanism may be involved [52, 53]. Cell apoptosis and inflammatory activation are the two major responses causing anti-DV NS1-induced endothelial cell damage. Autoantibody-induced endothelial dysfunction may also contribute to hepatic inflammation. Anti-DENV NS1 antibodies induced endothelial cell apoptosis by a Nitric Oxide (NO)-regulated pathway. Antibodies against DENV NS1 generated in mice cross-reacted with human endothelial cells and mouse vessel endothelium. After binding, mouse anti-NS1 Abs induced endothelial cell apoptosis in a caspase-dependent manner [56].

Molecular mimicry and autoimmunity have been demonstrated in Coxsackievirus and Epstein-Barr virus infections. Similarly, autoantibodies have also been associated as a significant factor involved in dengue virus pathogenesis [51]. In some recent studies, anti-NS1 antibodies were seen to result in autoimmunity, thus contributing to the development of severe dengue [8]. There are numerous self-antigens, which are known to exhibit sequence homology with DENV NS1 protein. Apart from DENV NS1, molecular mimicry has also been reported with other proteins such as DENV prM and E proteins, elucidating the cross-reactivity of the antibody against these antigens too [51]. Meanwhile, the autoantibodies, thus induced, can cross-react with several self-antigens, which include plasminogen, integrin, and platelet cells [8, 24, 57, 58]. Furthermore, stimulation of nitric oxide (NO) expression contributes to macrophage activation, endothelial cell lysis, and platelet aggregation causing apoptosis of endothelial cells, leading to thrombocytopenia, coagulopathy, and vascular leakage in severe dengue [59, 60].

Studies have shown that in patients of DHF/DSS, the levels of antiplatelet and anti-endothelial cell antibodies were found to be much higher than that in DF patients. Many studies have also demonstrated that IgM exhibits higher cross-reactivity than IgG against platelets and endothelial cells in DHF patients [51]. The role of anti-NS1 antibodies in autoimmunity during dengue virus infection has been proposed in vitro and in animal studies but its contribution in dengue patients is still not clearly proven due to lack of sufficient data.

### DENV Genome Variation and Subgenomic RNA

Severe dengue disease has been associated with all the four DENV serotypes. However, due to genetic differences between various DENV genotypes, each genotype has a difference in virulence as well as epidemic potential. This contributes to the development of severe clinical disease by few genotypes and strains [61, 62]. The year 1981 saw the emergence of the first outbreak of DHF in the Americas caused by DENV-2 Southeast Asian genotype, although the indigenous DENV-2 genotype was already present there for a long time. This evidence is sufficient to prove that DENV-2 Southeast Asian genotype is more virulent and replicates at a higher titer than the indigenous American DENV-2 genotype, thus leading to severe disease [63, 64].

In a study done by Cologna et al., it was demonstrated that the Southeast Asian genotype of DENV has a more efficient replication in the primary target cells, i.e., the immature dendritic cells which reside in the epidermis. This provides it a selective advantage over viruses of lower pathogenic potential, and it is known that higher viremias are more commonly associated with DHF [65, 66]. The generation of negative-strand RNA during early infection may determine higher virus output. This leads to an increase in genomic RNA and the number of secreted infectious viral particles. Thus, the Southeast Asian viruses are better adapted for production of virus progeny, independent of the number of cells they infect. This may explain the abilities of Southeast Asian viruses to replicate in the human body and infect the mosquito, thus initiating the cascade of events responsible for DHF in humans, and to be transmitted at much higher rates than the American counterpart in mosquitoes [66]. Phylogenetic studies on various genotypes of DENV-2 demonstrated that the native American genotype was associated with mild disease, whereas the introduction of Southeast Asian genotype in four different countries coincided with the appearance of DHF [67, 68]. In another study done in Peru, where from 1993 to 1997, no DHF cases were detected despite secondary infection rates of up to 75% and it was seen that there was no imported case of Southeast Asian genotype of DENV-2 [69]. Moreover, Anderson JR et al., demonstrated that viral replication in midgut was significantly higher in Southeast Asian genotype as compared to the American genotype infected *A. aegypti* mosquitoes. This higher replication in mosquitoes most likely contributes to increased dissemination of the particular genotype [64].

During the Cuban dengue epidemic in 1981 and 1997, there was an upward trend in case fatality rates towards the later part of the epidemic, thus leading to the conclusion that during the epidemic, infecting DENV might become more virulent and infective through transmission in hosts [8, 70–74]. Similar findings were also observed in the Townsville, Australia epidemic in 1992, thus proving the intra-serotypic evolutionary trend of epidemic DENV strains [72, 75]. In the DENV-2 outbreak in Cuba in 1997, analysis of six full genome sequences was carried out in a study and it was demonstrated that five consistent nucleotide changes in NS1, NS2A, and NS5 genes were present between early viruses when the number of cases was small, and late in the course of the epidemic when there was an increase in the number of total and DHF cases [72, 76]. Rodriguez-Roche R et al., performed a study on the 1997 Cuban outbreak of DENV infection. They demonstrated a month-by-month increase in clinical severity during secondary dengue infections. Some changes in the NS1 gene were observed that correlated with the observed rapid increase in disease severity, most notable of which was the substitution of threonine with serine at position 164 in NS1 protein. This change in NS1 gene may have resulted in increased viral fitness, due to changes in replication efficiency, thus leading to increased disease severity [73].

Chen HL et al., in their study done in Taiwan, examined viral sequences continuously at different time points during two outbreak of 2001 and 2002. They demonstrated that the 2002 viruses originated from a minor variant of the 2001 viruses. This proves that DENV may evolve via drift from existing minor variants in the population [72].

Duyen HT et al. reported that DENV-1 viremia levels were consistently higher in primary infection, in contrast to higher viremia levels of DENV-2 and DENV-3 observed in secondary infections. This suggests a distinctive behavior of DENV-1 serotype from others, which leads to high viral loads in the absence of pre-existing antibody. Meanwhile, in DENV-2 and DENV-3 infections, a heterologous immune response appears crucial for the development of high viral loads [47]. The same study also demonstrated that higher viral load on third day of illness was an independent predictor of higher haemoconcentration [47].

In a study by Yung C et al. in Singapore, it was demonstrated that infection with DENV-1 carries a higher risk of severe dengue as compared to DENV-2 [77]. This association was seen to be statistically significant. However, conflicting results were obtained in study by Vicente CR et al. in Brazil, who observed higher proportion of severe dengue cases among DENV-2 patients than that in DENV-1 [78]. The reasons for such contradictory findings in studies can be attributed to variable immune response due genetic differences in the study population.

Wang et al. revealed that in comparison to patients with DF, the levels of dengue virus RNA were significantly higher in DHF patients. The same study also demonstrated that dengue virus RNA remains in plasma for up to 6 days after defervescence in DHF patients, thus suggesting that viral RNA has a major role to play in the pathogenesis of severe dengue [79]. It might also indicate that high viral load persisting after defervescence may serve as a marker for DHF. These results explain the significance of viral factors and provide an insight into the pathogenesis of dengue [46, 79, 80].

Variation in DENV genome is not the only factor contributing to the pathogenesis of severe dengue, but the subgenomic flavivirus RNA (sfRNA) also has a major role to play in DENV replication in human host cells. sfRNA is generated when DENV RNA genome (11 kb) is incompletely broken down into small RNA (0.3–0.5 kb) by host exoribonuclease, during replication [8, 81–83]. This sfRNA accumulates and suppresses the host antiviral immune responses, particularly the type-1 IFN signaling [8, 84]. It may also cause immune evasion and lead to severe dengue by altering host mRNA stability [8, 85, 86].

Manokaran G et al., demonstrated that sfRNA has the ability to interact with proteins, thus inhibiting translation of interferon-stimulated genes. It was suggested that IFN-β suppression is most likely to be due to specific sfRNA–protein interactions. They also showed that the non-coding RNA of flaviviruses bind to and inactivate RNA-binding proteins, which are critical for innate immunity. Reduced IFN responses due to sfRNA may also lead to viral spread in susceptible cells in humans and to reach viremia levels sufficient for further mosquito-borne transmission [84]. In another study done by Chang RY et al. on Japanese Encephalitis virus (JEV), they demonstrated that sfRNA has a role in establishing persistent infection. They also showed that sfRNA reduced IFN-β promoter activity and IFN-β mRNA levels. It also leads to reduced phosphorylation of interferon regulatory factor-3 (IRF-3), the IFN-β upstream regulator, and blocked roughly 30% of IRF-3 nuclear localization. Moreover, JEV-infected sfRNA transfected cells produced 23% less IFN-β-stimulated apoptosis than mock-transfected groups did. These findings suggest that sfRNA plays a role against host cell antiviral responses, prevents cells from undergoing apoptosis, and thus contributes to viral persistence [87].

RNA interference (RNAi) is an important host defense mechanism against viral infections. In addition to the role of NS4B in interfering with RNAi pathways, sf RNA also has a role to play. It interacts with the Dicer protein and inhibits cleavage of dsRNA to small interfering RNA (siRNA) [88]. DENV sfRNA binds to **Tripartite motif-containing protein 25** (TRIM25) and inhibits the ubiquitination-mediated RIG-I activation, which subsequently suppresses IFN production [89].

### Role of Antibody-Dependent Enhancement (ADE)

There is enough evidence to prove that heterotypic secondary dengue infection has an increased risk for DHF/DSS than the primary infection [17]. Studies have revealed that infection in an infant with a serotype which is different than its infected mother, also carries an increased risk of severe dengue disease. This may occur in epidemic settings where mothers have experienced two or more dengue infections and their infants are at risk to severe and fatal dengue infections with any one of the four serotypes. This group comprises at least 5% of all children hospitalized for dengue in Southeast Asian countries [90, 91]. Some researchers have hypothesized that this increased risk is due to the antibodies produced as a result of primary infection, which are unable to neutralize the virus in the heterotypic secondary infection. This phenomenon has been called as antibody-dependent enhancement (ADE), which was first observed in 1973, when a comparatively rapid growth of DENV was seen in cultures of PBMCs obtained from dengue-immune individuals in contrast to non-immune individuals [92–95].

However, the mechanism of this was unknown. Later, this phenomenon was verified to be mediated by dengue antibodies, which were unable to neutralize the dengue virus [93, 96]. An enhancement of viral replication by more than 100-fold was observed when rhesus monkeys were passively immunized with anti-DENV antibodies before inoculation of virus [8, 97, 98].

The phenomenon of ADE in dengue virus infection was documented in the Cuban epidemic of 1977–79, 1981, and 1997. The predominant circulating serotype in these epidemics was DENV-1 in 1977–79 and DENV-2 in 1981 as well as 1997. During the 1997 outbreak, more than 200 cases of DHF/DSS were reported among adults above 15 years of age. Most of these cases were previously infected with DENV-1 serotype during the epidemic of 1977–79. Moreover, people who had been infected with DENV-1 during the 1977–79 outbreak followed by DENV-2 in 1997 had a 3–4-fold increased likelihood of developing severe disease than those secondarily infected with DENV-2 in 1981. This scenario can be explained by the presence of neutralizing heterotypic IgG antibodies in sufficient titers in 1981. The viral titers might have fallen by to the point where they no longer provided significant cross-protective immunity [99, 100].

The phenomenon of antibody-dependent enhancement is not only seen in dengue virus infection, but it has also been found to be associated with several other viral infections such as influenza, enteroviruses, etc. [8, 101, 102]. In spite of numerous studies, the exact mechanism of ADE remains incompletely understood. A subsequent heterotypic dengue infection after a primary infection may lead to the cross-reactive non-neutralizing pre-existing antibodies binding to the virus but not being able to neutralize it, thus forming virion-antibody immune complexes. This facilitates the uptake of virus-immune complexes into the phagocytes after being recognized by Fcγ receptor, leading to amplification in viral replication. This increase in viral load starts an immunopathogenic cascade, ultimately leading to vascular leak and manifestations of severe dengue infection [13, 103].

In addition to the Fcγ receptor-mediated amplification in viral replication, the other mechanism proposed for the enhancement of viral replication is known as intrinsic ADE. This hypothesis suggests that internalization of DENV, through Fcγ receptor, inhibits antiviral genes by suppressing the type-1 IFN-mediated antiviral responses. At the same time, enhanced production of IL-10 skews the immune response towards T_H_-2, which has limited antiviral effect and is unable to clear the virus [51, 104, 105]. Furthermore, Fcγ receptor-mediated ADE causes release of cytokines from immune cells leading to vascular endothelial cell dysfunction and increased vascular permeability [106, 107].

Katzelnick et al. performed a cohort study in pediatric population in Nicaragua and used multiple statistical approaches to analyze the observations. The association between pre-existing anti-DENV binding antibodies and dengue disease severity was evaluated in the study. This study was conducted in children aged 2–14 years and 8002 cohort participants (6684 included in study who had at least one DENV-Ab titer measurement) were included. Anti-DENV antibody (DENV-Ab) titres were measured in paired annual samples using an inhibition ELISA (iELISA) method. As the children reached 15 years of age, they were removed from the study and new 2-year-old children were included. A peak enhancement titer was observed, which implied that DENV infection was enhanced at a specific antibody concentration (DENV-Ab titer 1:21 to 1:80). Antibody concentrations lower than that specific level failed to enhance the infection, whereas a higher antibody concentration was able to neutralize the virus [103]. The hazard of severe dengue was observed to be similar in children with no (DENV-naïve) or high (> 1:1280) DENV-Ab titres. Further, severe secondary dengue cases each with five matched controls were studied and a similar peak enhancement titer was observed at DENV-Ab titres of 1:21 to 1:80, with reduced odds ratios at lower and higher DEN-Ab titres. This finding was corroborated by the safety and efficacy trials of tetravalent dengue vaccine (CYD-TDV), which showed that the vaccine protected partially against severe dengue disease and hospitalization for at least 5 years in individuals who had previous exposure to dengue before the vaccination. In contrast, higher risk of severe disease was observed in vaccinated individuals who had not been exposed to dengue previously [108].

Some studies have also shown that the ‘enhancing vaccine concept’ is not consistent and sustained [109, 110]. In spite of this, regional health organizations have concluded that it is premature to recommend application of the vaccine in a routine vaccination program because of the probable risk of severe illness through immune potentiation in age groups with higher seronegative proportions [110, 111].

Till date, demonstration of protective neutralizing antibodies to DENV was the only interest to dengue vaccine manufacturers. However, a reassessment of this assumption needs to be carried out due to the recent evidence of dengue vaccine mimicking a primary infection, leading to a possible vaccine-enhanced dengue disease in subsequent infection [109]. A vaccine that induces antibody titers at, or near, the peak enhancement titer, may place the vaccinees at a greater risk of severe dengue than if they had never been vaccinated [103, 112].

### Role of T Cells

Various studies have implied that cross-reactive T cells, which are active during secondary heterotypic infections, play a role in mediating the pathogenesis of DHF [16]. The T cells, which are activated due to antigen exposure, are more abundant in early convalescence in patients with severe dengue as compared to those with milder disease [113].

The CD8+ T cell epitopes are primarily found in the non-structural proteins NS3 and NS5 [113, 114]. Activated CD8+ T cells in a heterologous secondary DENV infection showed high cross-reactivity and were found to be inefficient at clearing the newly infective virus serotype, owing to low avidity [30]. In DHF patients, higher levels of cytokines and chemokines are produced by activated DENV-specific cross-reactive T cells, which suggests a positive association between cross-reactive T cell activation and the severity of dengue-associated disease [39, 115, 116].

IL-10 may play an important in DENV pathogenesis, as it exhibits pleiotropic effects in immunoregulation and inflammation. It has been seen that IL-10 levels are higher in DHF/DSS patients as compared to DF patients. There is some speculation regarding the relationship between IL-10 and viral replication, and the most probable role of IL-10 may be due to inhibition of antiviral IFN response.

The main cells which produce IL-10 are monocytes/macrophages, type 2 T-helper cells, and CD4^+^CD25^+^Foxp3^+^ regulatory T cells. In has been observed that in acute dengue infection, there is presence of increased frequencies of CD4^+^CD25^high^ regulatory T cells. It has also been demonstrated that IL-10 is induced only in ADE infection, but not in DENV infection alone. It implies that DENV and ADE co-regulate IL-10 production, which is increased DHF/DSS patients. Extrinsic ADE infection contributes to a high rate of viral infection in Fcγ receptor-bearing cells, whereas the intrinsic ADE effect via IL-10 suppresses the activation of the IFN-mediated antiviral response.

NF-κB is critical for TLR-mediated antiviral IFN responses, pro-inflammatory activation, production of IL-2, IL-12, TNF-α, and IFN-γ, and expression of MHC class II antigens and co-stimulatory molecules, which is blocked by IL-10. Increased production of IL-10 also inhibits the action of antiviral NK cells during the immune response to DENV infection. These mechanisms may be responsible for prolonging viral infection and inhibiting IL-10 might facilitate antiviral response. High titres of viremia, caused by the ADE of DENV infection, determine the frequency of DHF/DSS progression. In addition to the involvement of extrinsic ADE-mediated viral infection, delayed viral clearance mediated through IL-10 immunosuppression may be involved in DENV pathogenesis. IL-10 may cause lymphocyte dysfunction through the suppression of the T cell proliferative response to mitogens, which occurs in dengue patients during the early stages of infection. Moreover, increased IL-10 levels have been associated with thrombocytopenia and serum levels of AST and ALT. Elevated IL-10 levels in sera of severe dengue patients, leads to T cell apoptosis resulting in reduced viral clearance and impaired antiviral response [16, 117, 118].

It has been demonstrated that dysregulated cytokine cascade has a vital role in the pathogenesis of DHF [119]. The key cytokines associated with the development of DHF include the shift from Th1-type response in DF to Th2-type cytokine response in DHF, which leads to increased levels of IL-10 and IL-14 [7]. The key player in pathogenesis of DHF appears to be a unique cytokine produced by CD4+ T cells, known as the cytotoxic factor (hCF) [120, 121]. hCF induces macrophages to produce free radicals, nitrite and reactive oxygen species, which directly upregulate the production of pro-inflammatory cytokines IL-1, TNF-α, IL-8 and H_2_O_2_ in macrophages, besides killing the target cells via apoptosis. This shifts a Th1-dominant response to Th2-biased immune response, leading to manifestations of severe dengue disease [121].

The clearing of DENV-infected cells by activated T cells is dependent on the avidity of the T cell receptor (TCR) for the HLA-peptide complex. During acute secondary infection with heterologous DENV, highly cross-reactive CD8^+^ T cells with high avidity are preferentially activated, which produce high levels of pro- and anti-inflammatory cytokines such as TNF-α, IFN-ϒ and IL-13, but lower levels of IL-10. However, these high avidity cross-reactive CD8^+^ T cells die through apoptosis, the reasons for which are not completely clear. Alternatively, the low-avidity cross-reactive CD8^+^ T cells get preferentially expanded, which react differently to heterologous epitopes than to homologous epitopes by producing high levels of pro-inflammatory cytokines, but they lose their cytolytic activity [30]. This results in delayed virus clearance, which subsequently causes prolonged activation of such cross-reactive CD8^+^ T cells leading to increased production of TNF-α and IL-6. This phenomenon, which has been called ‘Original antigenic sin (OAS)’ ultimately affects the vascular permeability and progression to severe manifestations of DHF/DSS [30, 122].

Overall, the CD4+ T cell responses have been less well characterized in DENV infection. In contrast to CD8+ T cell responses, DENV-specific CD4+ T cells epitopes are predominantly located in structural proteins like envelope and capsid, in addition to NS1 protein [114]. Evidence also exists that the cytokine response of cross-reactive CD4+ T cells might be altered by the sequential infection with different DENV serotypes, leading to increased levels of pro-inflammatory cytokines that contributes to a detrimental immune response causing severe dengue [30].

The role of immune regulatory mechanisms of the immune system, which are CD4+ FoxP3 expressing regulatory T cells (T_regs_), has also been investigated by some researchers. Regulatory T cells are known to be a double-edged sword. A balance between Th1 and Th2 is important for the control of immune response to microorganisms. In DENV infection, the T_reg_ cells may suppress protective Th1 response and enhance the Th2 response leading to increase in infection-induced immunopathology. Loss of suppressor of cytokine signaling (SOCS3) in T helper cells results in reduced immune responses and hyperproduction of IL-10 and TGF-β [121, 123]. The T_reg_ cells also suppress antigen-specific antibody production, thus preventing increased replication of virus mediated by ADE and immune complex formation [121]. Tian Y et al., in their study analyzed that the ratio of T_reg_ cells to effector T cells is significantly higher during acute dengue infection in patients with mild disease, but not in those with severe disease [124]. Both the Treg cells and Th2 cells secrete IL-10 and TGF-β, causing a disturbance in the fine balance of different cytokines ultimately leading to ‘cytokine storm’ and severe dengue disease [121]. A defect in T_reg_ cells, leading to defective immune suppression, may be a contributing factor to severe clinical disease. A high T_regs_/effector T cell ratio was found to be seen in milder clinical disease, suggesting that impaired functional regulatory T cell responses may lead to severe dengue disease [125]. The proven role of Treg cells in severe dengue disease has prompted researchers to think about manipulation of this subset of T cells in future for use in patients for treatment by enhancing/depressing their suppressor function.

Summary of few studies on various viral factors implicated in pathogenesis of dengue virus infection is shown in Table 1.

**Table 1:** Summary of various factors implicated in pathogenesis of dengue virus infection

Role of NS1 antigen				
Reference	Year	Study design	Human/Animal/ in-vivo/in-vitro	Role in Pathogenesis
Modhiran N et al.	2015	Experimental	In-vitro	DENV NS1 disrupts the endothelial cell monolayer
Suresh R et al.	2016	Experimental	Animal	Positive correlation between DENV NS1 levels and C5b-C9 complex formation
Paranavitane SA et al.	2014	Prospective	Human	DENV NS1 antigen persists for longer duration in patients of vascular leakage
Chen HR et al.	2016	Experimental	Animal	NS1-induced MIF secretion and autophagy may contribute to vascular leakage in severe dengue
Cruz-Hernandez et al.	2013	Transverse retrospective	Human	Higher NS1 levels in patients suffering from DHF in DENV-1
**Anti-NS1 antibodies and Autoimmunity**				
Lin CF et al.	2005	Experimental	Animal, Human	DENV NS1 antibodies stimulate the release of multiple inflammatory factors
Sun DS et al.	2007	Experimental	Human, Animal	Induce cellular damage by complement-mediated lysis
**DENV genome variation**				
Vasilakis N et al.	2007	Experimental	Animal, Human in-vitro & in-vivo	Genotype-specific virulence
Ritchie SA et al. Chen HL et al.	2013 2008	Epidemiological Analytical	Human Human	Intra-epidemic increase in virulence
Duyen HT et al.	2011	Cohort	Human	Consistently higher DENV-1 viremia levels in primary infection Higher viremia levels of DENV-2 and DENV-3 in secondary infections.
Fox A et al.	2011	Cohort	Human	Significantly lower viremia in DENV-2 than that of DENV-1 on fifth day of illness
Manokaran G et al. Moon SL et al.	2015 2012	Experimental Experimental	In-vitro In-vitro	Suppression of host antiviral immune responses by sfRNA
**Role of Antibody-Dependent Enhancement**				
Goncalvez Ap et al.	2007	Experimental	Animal	Viral replication rate enhanced in passively immunized rhesus monkeys
Guzman MG et al.	2007	Retrospective	Human	ADE demonstrated during Cuban outbreaks in study groups
Katzelnick LC et al.	2017	Cohort	Human	Observed peak enhancement titer
Sridhar S et al.	2018	Cohort	Human	ADE observed in naïve individuals vaccinated with CTD-TDV vaccine
Ubol S et al.	2010	Prospective and experimental	Human in-vivo and in-vitro	Inhibits antiviral genes and enhancing the production of IL-10.
Brown MG et al.	2011	Experimental	In-vitro	Causative role of vascular endothelial cell dysfunction and increased vascular permeability
**Role of Cross-reactive T cells**				
Simmons CP et al.	2015	Review article	–	Antigen activated T cells more abundant in patients with severe dengue
Martina BEE et al.	2009	Review article	–	Activated CD8+ and CD4+ cells secondary DENV infection inefficient at killing infected cells
Malavige G et al.	2011	Review article	–	High T_regs_/effector T cell ratio associated with milder clinical disease

### Role of Host Genetic Factors

Apart from various viral factors implicated in the pathogenesis of DENV infection, host genetic factors also play an important role. One of the host genetic factors which have a role in DENV pathogenesis is of the Human Leucocyte Antigen (HLA). Many HLA class-I alleles have been observed to have an association with severe dengue in secondary infections, which suggests the importance of existing primed memory HLA Class-I restricted cross-reactive T cells. However, HLA class-II (especially DRB1 alleles) has been shown to exert a protective effect on DENV infection and disease severity [126, 127]. It has also been shown that HLA-DR4 plays a protective role against DHF [127]. Moreover, TNF-α, which is located on the HLA Class-III sub-region of Major histocompatibility complex (MHC), is known to be upregulated in DHF [126, 128]. It was seen that polymorphism in the promoter region of TNF-α gene, −308A allele, is a risk factor for the development of DHF in South American patients but not in Southeast Asian patients [129]. There were a lot of differences observed in HLA allele frequency, with A*0203, B*52 and DRB1*04 acting as protective factors, while B*51, DQB1*0302, and TNF-α- LTA haplotype were observed as susceptible factors in Thai and Mexican studies [126].

Apart from HLA genes having a role in host genetic susceptibility to DF and DHF, genetic polymorphisms in several non-HLA genes also play a role, which include Fcγ receptor II (FcγRII, CD32), Vitamin D receptor (VDR), Human Platelet Antigen (HPA), Transporter associated with Antigen Processing (TAP), and few cytokine polymorphisms. Homozygotes for arginine variant at position 131 of the FcγRII gene were seen to be protective against DSS in a Vietnamese population [130]. VDR polymorphism analysis by Loke H et al., demonstrated that the C allele at position 352 was resistant to DSS [131]. In an Indian study done by Soundravally R et al. on the role of TAP and HPA gene polymorphisms in DHF and DSS, it was demonstrated that heterozygous pattern at the TAP1 333 locus and HPA1a/1a and HPA2a/2b genotypes confer susceptibility to DHF and the HPA1a/1b genotype was determined to be a genetic risk factor for DSS [132].

IL-10 (−1082/−819/−592) ACC/ATA haplotype was shown to be significantly associated with the development of DHF in a study carried out by Perez AB et al., in Cuban population [129].

In a study done by Chen et al. in Taiwan, it was demonstrated that the risk of DHF is twice in individuals carrying TGFβ1–509 CC genotype. The risk further increased if the individual also had CTLA-4 + 49 G allele [126].

With these findings, it can be conclusively said that the associations between host genetics, DENV infection and clinical outcome are complex, and require further exploration and in-depth studies.

### Recent Insights into Dengue Pathogenesis and Biomarkers

Identification of markers for disease severity or increase risk of progression to more severe forms of the disease is a major interest in dengue research. Most of the studies have been done on immunological biomarkers of severe dengue infections, which include IL-7, IL-8, IL-10, TGF-β, TNF-α, IFN-ϒ, etc. The elevated levels of cytokines in severe dengue make them an excellent predictor of severity of dengue infection. Studies have shown that cytokine rise at presentation may indicate whether a patient is likely to develop severe dengue or not [133, 134]. NS1-mediated cytokine release can be inhibited by TLR4-antagonist LPS-Rhodobacter sphaeroides, a gram-negative facultative photosynthetic bacterium which can lead to targeted therapy in future [22]. Another study has shown interest in the use of serum chymase levels as a predictive biomarker of DHF. The study revealed that acute dengue infection patients who have high levels of serum chymase consistently are at a greater risk of severe disease [135]. Research on the utilization of microparticles (MPs) as predictive markers for severe dengue infections has also revealed promising results. There is evidence of a significant role of MPs in dengue pathogenesis. Decreased platelet-derived MPs were associated with bleeding tendency, whereas increased levels of red blood cell-derived MPs (RMPs) correlated with more severe disease. It has been seen that increased levels of red blood cell-derived MPs (RMPs) directly correlated with severe dengue [136].

MicroRNAs (miRNA) are endogenous non-coding single-stranded RNA molecules made of approximately 22 nucleotides and play a vital role in gene regulation of organism, as their abnormal expression is related with the occurrence and developments of diseases [137]. In a study done by Ouyang X et al., it was demonstrated that 53 aberrantly expressed miRNA were detected in sera of DENV-infected patients, which indicates that these patients show a broad profile of serum miRNA dysfunction. miRNA PCR array assays have revealed differential expression of few upregulated and few downregulated miRNAs in sera levels in dengue patients. The miRNAs which were identified as promising serum indicators for dengue infection are serum hsa-miR-21-5p, hsa-miR-146a-5p, hsa-miR-590-5p, hsa-miR-188-5p, and hsa-miR-152-3p.

DENV infection induces the expression of miR-146a in primary monocytes in vitro, which plays an important role in regulating innate immune, inflammatory response, and virus infection. MiR-21 plays a vital role in the resolution of inflammation and negative regulation of pro-inflammatory response. MiR-188 and miR-152 are apparently associated with the inhibition of cell proliferation and invasion, whereas miR-21 and miR-590 may promote cell proliferation [137, 138]. In silico analyses have revealed that DENV can hijack a host cell’s machinery and evade antiviral immune response by miRNA mechanisms [137, 139]. However, the roles of these dysfunctions of serum miRNA in the pathological process of DENV infection need to be defined in vivo.

Moreover, miRNAs have been observed to play major role in the regulation of physiological and pathological states. The secreted miRNAs can mediate cell-to-cell communications. The involvement of miRNAs in inflammation and cell proliferation has been shown to be significant. The miRNAs which have been identified to have a potential role as non-invasive molecular markers for detecting DENV infection include hsa-miR-21, has-miR-146a-5p, hsa-miR-590-5p, hsa-miR-188-5p, and hsa-miR-152-3p [137]. However, no conclusive biological functions in pathological process of dengue infection could be attributed to these miRNAs. In view of this, further research is needed to arrive at a conclusion of use of these biomarkers as early predictors of dengue disease severity and give us more insight into their role in pathogenesis of dengue virus infection and severe dengue.

## Conclusion

The exact mechanisms responsible for severe manifestations of dengue virus disease are not yet fully elucidated and are likely to be multifactorial. DENV infection can have diverse presentations in different hosts, which cannot be explained by a single hypothesis alone. The recent results of the efficacy and safety trials of the CYD-TDV tetravalent dengue vaccine have reminded the research community that there is still a lot to learn with regard to understanding the pathogenesis and expression of protective immunity to DENV.

The emerging picture is that multiple factors including prior immunity, viral load, NS1, anti-NS1 antibodies, infecting serotype, and genotype may contribute to the severity of dengue infection, but the nature of these interactions remains unclear and substantial gaps still exist in the comprehensive understanding of the pathogenesis of dengue virus infections.

The complete understanding of the pathogenesis of dengue virus infection is an ever-evolving field of research. An insight into various unexplored or less explored areas in this field will help in developing antivirals and effective vaccine for dengue. There are a lot of promising areas in this field which could be progressed like animal experimentation, examining the direct effect of dengue virus NS1 antigen on capillary permeability and also the association of NS1 levels with disease severity. This will help us to understand the role of NS1 antigen and its association with disease severity. It will also help us understand that whether NS1 level estimation early in the course of disease can be used as a biomarker for predicting severe dengue disease. Moreover, further research needs to be done on the role of miRNAs and its biological role in causing the pathological manifestations of dengue virus infection.

## References

[1] Mutheneni SR, Morse AP, Caminade C, Upadhyayula SM (2017). Dengue burden in India: recent trends and importance of climatic parameters. Emerg Microbes Infect.

[2] Bhatt S, Gething PW, Brady OJ, Messina JP, Farlow AW, Moyes CL (2013). The global distribution and burden of dengue. Nature..

[3] Hussain T, Jamal M, Rehman T, Andleeb S (2015). Dengue: pathogenesis, prevention and treatment – a mini review. Adv Life Sci.

[4] Hawley WA, Reiter P, Copeland RS, Pumpuni CB, Craig GB (1987). Aedes albopictus in North America: probable introduction in used tires from northern Asia. Science..

[5] Holmes EC (1998). Molecular epidemiology and evolution of emerging infectious diseases. Br Med Bull.

[6] Mustafa MS, Rasotgi V, Jain S, Gupta V (2015). Discovery of fifth serotype of dengue virus (DENV-5): a new public health dilemma in dengue control. Med J Armed Forces India.

[7] Chaturvedi UC, Agarwal R, Elbishbishi EA, Mustafa AS (2000). Cytokine cascade in dengue hemorrhagic fever: implications for pathogenesis. FEMS Immunol Med Microbiol.

[8] Pang X, Zhang R, Cheng G (2017). Progress towards understanding the pathogenesis of dengue hemorrhagic fever. Virol Sin.

[9] Hermann LL, Gupta SB, Manoff SB, Kalayanarooj S, Gibbons RV, Coller B-AG (2015). Advances in the understanding, management, and prevention of dengue. J Clin Virol.

[10] World Health Organization (1997) Dengue haemorrhagic fever diagnosis, treatment, p.pdf [Internet]. [cited 2018 July 3]. Available from: http://apps.who.int/iris/bitstream/handle/10665/41988/9241545003_eng.pdf;jsessionid=839B7D324058697247721C394B7C052F?sequence=1

[11] Special Programme for Research and Training in Tropical Diseases and World Health Organization (2009) Dengue guidelines for diagnosis, treatment, preve.pdf [Internet]. [cited 2018 July 3]. Available from: http://www.who.int/tdr/publications/documents/dengue-diagnosis.pdf

[12] Halstead SB (2012). Controversies in dengue pathogenesis. Paediatr Int Child Health.

[13] Sellahewa KH (2013). Pathogenesis of dengue haemorrhagic fever and its impact on case management [Internet]. International Scholarly Research Notices. 2013 [cited 2019 Apr 30]. Available from: https://www.hindawi.com/journals/isrn/2013/571646/

[14] Green S, Rothman A (2006). Immunopathological mechanisms in dengue and dengue hemorrhagic fever. Curr Opin Infect Dis.

[15] Weaver SC, Vasilakis N (2009). Molecular evolution of dengue viruses: contributions of phylogenetics to understanding the history and epidemiology of the preeminent arboviral disease. Infect Genet Evol.

[16] Mathew A, Rothman AL (2008). Understanding the contribution of cellular immunity to dengue disease pathogenesis. Immunol Rev.

[17] Guzman MG, Alvarez M, Halstead SB (2013). Secondary infection as a risk factor for dengue hemorrhagic fever/dengue shock syndrome: an historical perspective and role of antibody-dependent enhancement of infection. Arch Virol.

[18] Simmons CP (2010) Chapter 127 – Dengue. In: Cohen J, Opal SM, Powderly WG, eds. Infectious diseases (3rd ed.). [Internet]. Content Repository Only!, London, p 1253–1256. [cited 2020 Aug 29]. Available from: http://www.sciencedirect.com/science/article/pii/B9780323045797001271

[19] Anderson KB, Gibbons RV, Cummings DAT, Nisalak A, Green S, Libraty DH (2014). A shorter time interval between first and second dengue infections is associated with protection from clinical illness in a school-based cohort in Thailand. J Infect Dis.

[20] Nimmannitya S, Halstead SB, Cohen SN, Margiotta MR (1969). Dengue and chikungunya virus infection in man in Thailand, 1962-1964. I. Observations on hospitalized patients with hemorrhagic fever. Am J Trop Med Hyg.

[21] Halstead SB, Nimmannitya S, Cohen SN (1970). Observations related to pathogenesis of dengue hemorrhagic fever. IV. Relation of disease severity to antibody response and virus recovered. Yale J Biol Med.

[22] Halstead S (2019) Recent advances in understanding dengue. F1000Research [Internet]. [cited 2020 Jan 4];8. Available from: https://www.ncbi.nlm.nih.gov/pmc/articles/PMC6676504/10.12688/f1000research.19197.1 PMC6676504 31448083

[23] Burke DS, Nisalak A, Johnson DE, Scott RM (1988). A prospective study of dengue infections in Bangkok. Am J Trop Med Hyg.

[24] Chuang Y-C, Lin J, Lin Y-S, Wang S, Yeh T-M (2016). Dengue virus nonstructural protein 1-induced antibodies cross-react with human plasminogen and enhance its activation. J Immunol.

[25] Chuang Y-C, Wang S-Y, Lin Y-S, Chen H-R, Yeh T-M (2013). Re-evaluation of the pathogenic roles of nonstructural protein 1 and its antibodies during dengue virus infection. J Biomed Sci.

[26] Srikiatkhachorn A, Kelley JF (2014). Endothelial cells in dengue hemorrhagic fever. Antivir Res.

[27] Modhiran N, Watterson D, Muller DA, Panetta AK, Sester DP, Liu L (2015). Dengue virus NS1 protein activates cells via Toll-like receptor 4 and disrupts endothelial cell monolayer integrity. Sci Transl Med.

[28] Malavige GN, Ogg GS (2017). Pathogenesis of vascular leak in dengue virus infection. Immunology.

[29] Glasner DR, Puerta-Guardo H, Beatty PR, Harris E (2018). The good, the bad, and the shocking: the multiple roles of dengue virus nonstructural protein 1 in protection and pathogenesis. Annu Rev Virol.

[30] Martina BEE, Koraka P, Osterhaus ADME (2009). Dengue virus pathogenesis: an integrated view. Clin Microbiol Rev.

[31] Suresh R, Chandrasekaran P, Sutterwala FS, Mosser DM (2016). Complement-mediated “bystander” damage initiates host NLRP3 inflammasome activation. J Cell Sci.

[32] Ubol S, Masrinoul P, Chaijaruwanich J, Kalayanarooj S, Charoensirisuthikul T, Kasisith J (2008). Differences in global gene expression in peripheral blood mononuclear cells indicate a significant role of the innate responses in progression of dengue fever but not dengue hemorrhagic fever. J Infect Dis.

[33] Conde JN, Silva EM, Barbosa AS, Mohana-Borges R (2017) The Complement System in Flavivirus Infections. Front Microbiol [Internet]. [cited 2020 Aug 29];8. Available from: https://www.frontiersin.org/articles/10.3389/fmicb.2017.00213/full 10.3389/fmicb.2017.00213 PMC5306369 28261172

[34] Avirutnan P, Malasit P, Seliger B, Bhakdi S, Husmann M (1998). Dengue virus infection of human endothelial cells leads to chemokine production, complement activation, and apoptosis. J Immunol Baltim Md 1950.

[35] Puerta-Guardo H, Glasner DR, Harris E (2016). Dengue virus NS1 disrupts the endothelial Glycocalyx, leading to hyperpermeability. PLOS Pathog.

[36] Chen H-R, Chuang Y-C, Lin Y-S, Liu H-S, Liu C-C, Perng G-C (2016). Dengue virus nonstructural protein 1 induces vascular leakage through macrophage migration inhibitory factor and autophagy. PLoS Negl Trop Dis.

[37] Paranavitane SA, Gomes L, Kamaladasa A, Adikari TN, Wickramasinghe N, Jeewandara C, et al. (2014) Dengue NS1 antigen as a marker of severe clinical disease. BMC Infect Dis [Internet]. [cited 2018 Sep 17]; 14. Available from: https://www.ncbi.nlm.nih.gov/pmc/articles/PMC4222370/10.1186/s12879-014-0570-8 PMC4222370 25366086

[38] Adikari TN, Gomes L, Wickramasinghe N, Salimi M, Wijesiriwardana N, Kamaladasa A (2016). Dengue NS1 antigen contributes to disease severity by inducing interleukin (IL)-10 by monocytes. Clin Exp Immunol.

[39] Malavige GN, Huang L-C, Salimi M, Gomes L, Jayaratne SD, Ogg GS (2012). Cellular and cytokine correlates of severe dengue infection. PLoS One.

[40] Duong V, Ly S, Lorn Try P, Tuiskunen A, Ong S, Chroeung N (2011). Clinical and virological factors influencing the performance of a NS1 antigen-capture assay and potential use as a marker of dengue disease severity. PLoS Negl Trop Dis.

[41] Avirutnan P, Punyadee N, Noisakran S, Komoltri C, Thiemmeca S, Auethavornanan K (2006). Vascular leakage in severe dengue virus infections: a potential role for the nonstructural viral protein NS1 and complement. J Infect Dis.

[42] Kurosu T, Chaichana P, Yamate M, Anantapreecha S, Ikuta K (2007). Secreted complement regulatory protein clusterin interacts with dengue virus nonstructural protein 1. Biochem Biophys Res Commun.

[43] Muller DA, Young PR (2013). The flavivirus NS1 protein: molecular and structural biology, immunology, role in pathogenesis and application as a diagnostic biomarker. Antivir Res.

[44] Rastogi M, Sharma N, Singh SK (2016) Flavivirus NS1: a multifaceted enigmatic viral protein. Virol J [Internet]. [cited 2020 Aug 29];13. Available from: https://www.ncbi.nlm.nih.gov/pmc/articles/PMC4966872/10.1186/s12985-016-0590-7 PMC4966872 27473856

[45] Mackenzie JM, Jones MK, Young PR (1996). Immunolocalization of the dengue virus nonstructural glycoprotein NS1 suggests a role in viral RNA replication. Virology..

[46] Libraty DH, Young PR, Pickering D, Endy TP, Kalayanarooj S, Green S (2002). High circulating levels of the dengue virus nonstructural protein NS1 early in dengue illness correlate with the development of dengue hemorrhagic fever. J Infect Dis.

[47] Duyen HTL, Ngoc TV, Ha DT, Hang VTT, Kieu NTT, Young PR (2011). Kinetics of plasma viremia and soluble nonstructural protein 1 concentrations in dengue: differential effects according to serotype and immune status. J Infect Dis.

[48] de la Cruz-Hernández SI, Flores-Aguilar H, González-Mateos S, López-Martinez I, Alpuche-Aranda C, Ludert JE (2013). Determination of viremia and concentration of circulating nonstructural protein 1 in patients infected with dengue virus in Mexico. Am J Trop Med Hyg..

[49] Fox A, Le NMH, Simmons CP, Wolbers M, Wertheim HFL, Pham TK (2011). Immunological and viral determinants of dengue severity in hospitalized adults in Ha Noi, Viet Nam. PLoS Negl Trop Dis.

[50] Lin C-F, Chiu S-C, Hsiao Y-L, Wan S-W, Lei H-Y, Shiau A-L (2005). Expression of cytokine, chemokine, and adhesion molecules during endothelial cell activation induced by antibodies against dengue virus nonstructural protein 1. J Immunol.

[51] Wan S-W, Lin C-F, Yeh T-M, Liu C-C, Liu H-S, Wang S (2013). Autoimmunity in dengue pathogenesis. J Formos Med Assoc.

[52] Lin C-F, Wan S-W, Chen M-C, Lin S-C, Cheng C-C, Chiu S-C (2008). Liver injury caused by antibodies against dengue virus nonstructural protein 1 in a murine model. Lab Invest.

[53] Huerre MR, Lan NT, Marianneau P, Hue NB, Khun H, Hung NT (2001). Liver histopathology and biological correlates in five cases of fatal dengue fever in Vietnamese children. Virchows Arch Int J Pathol.

[54] Mehta V, Verma R, Garg R, Malhotra H, Sharma P, Jain A (2017). Study of interleukin-6 and interleukin-8 levels in patients with neurological manifestations of dengue. J Postgrad Med.

[55] Priyadarshini D, Gadia RR, Tripathy A, Gurukumar KR, Bhagat A, Patwardhan S, et al (2010) Clinical findings and pro-inflammatory cytokines in dengue patients in Western India: a facility-based study. PLoS ONE [Internet]. [cited 2020 Aug 29];5(1). Available from: https://www.ncbi.nlm.nih.gov/pmc/articles/PMC2806829/10.1371/journal.pone.0008709 PMC2806829 20090849

[56] Lin C-F, Lei H-Y, Shiau A-L, Liu H-S, Yeh T-M, Chen S-H (2002). Endothelial cell apoptosis induced by antibodies against dengue virus nonstructural protein 1 via production of nitric oxide. J Immunol.

[57] Falconar AK (1997). The dengue virus nonstructural-1 protein (NS1) generates antibodies to common epitopes on human blood clotting, integrin/adhesin proteins and binds to human endothelial cells: potential implications in haemorrhagic fever pathogenesis. Arch Virol.

[58] Rachman A, Harahap AR, Widhyasih RM (2013). The role of anti-dengue virus NS-1 and anti-protein disulfide isomerase antibodies on platelet aggregation in secondary dengue infection. Acta Med Indones.

[59] Sun D-S, King C-C, Huang H-S, Shih Y-L, Lee C-C, Tsai W-J (2007). Antiplatelet autoantibodies elicited by dengue virus non-structural protein 1 cause thrombocytopenia and mortality in mice. J Thromb Haemost.

[60] Wan S-W, Lu Y-T, Huang C-H, Lin C-F, Anderson R, Liu H-S (2014). Protection against dengue virus infection in mice by administration of antibodies against modified nonstructural protein 1. PLoS One.

[61] Ubol S, Halstead SB (2010). How innate immune mechanisms contribute to antibody-enhanced viral infections. Clin Vaccine Immunol.

[62] Mammen MP, Lyons A, Innis BL, Sun W, McKinney D, Chung RCY (2014). Evaluation of dengue virus strains for human challenge studies. Vaccine..

[63] Vasilakis N, Shell EJ, Fokam EB, Mason PW, Hanley KA, Estes DM (2007). Potential of ancestral sylvatic dengue-2 viruses to re-emerge. Virology..

[64] Anderson JR, Rico-Hesse R (2006). Aedes aegypti vectorial capacity is determined by the infecting genotype of dengue virus. Am J Trop Med Hyg..

[65] Libraty DH, Endy TP, Houng H-SH, Green S, Kalayanarooj S, Suntayakorn S (2002). Differing influences of virus burden and immune activation on disease severity in secondary dengue-3 virus infections. J Infect Dis.

[66] Cologna R, Armstrong PM, Rico-Hesse R (2005). Selection for virulent dengue viruses occurs in humans and mosquitoes. J Virol.

[67] Leitmeyer KC, Vaughn DW, Watts DM, Salas R, Villalobos I, de Chacon (1999). Dengue virus structural differences that correlate with pathogenesis. J Virol.

[68] Rico-Hesse R, Harrison LM, Salas RA, Tovar D, Nisalak A, Ramos C (1997). Origins of dengue type 2 viruses associated with increased pathogenicity in the Americas. Virology..

[69] Watts DM, Porter KR, Putvatana P, Vasquez B, Calampa C, Hayes CG (1999). Failure of secondary infection with American genotype dengue 2 to cause dengue haemorrhagic fever. Lancet.

[70] Ritchie SA, Pyke AT, Hall-Mendelin S, Day A, Mores CN, Christofferson RC (2013). An explosive epidemic of DENV-3 in Cairns, Australia. PLOS ONE.

[71] Guzmán MG, Kourí G, Halstead SB (2000). Do escape mutants explain rapid increases in dengue case-fatality rates within epidemics?. Lancet.

[72] Chen H-L, Lin S-R, Liu H-F, King C-C, Hsieh S-C, Wang W-K (2008). Evolution of dengue virus type 2 during two consecutive outbreaks with an increase in severity in southern Taiwan in 2001-2002. Am J Trop Med Hyg..

[73] Rodriguez-Roche R, Sanchez L, Burgher Y, Rosario D, Alvarez M, Kouri G (2011). Virus role during intraepidemic increase in dengue disease severity. Vector Borne Zoonotic Dis.

[74] Halstead SB (2014) Intraepidemic increases in dengue disease severity: applying lessons on surveillance & transmission. In: Clinical insights: dengue: transmission, diagnosis & surveillance [Internet]. Future Medicine Ltd; [cited 2020 Jan 4]. p 83–101. Available from: https://www.futuremedicine.com/doi/10.2217/ebo.13.741

[75] Streatfield R, Bielby G, Sinclair D (1993). A primary dengue 2 epidemic with spontaneous haemorrhagic manifestations. Lancet.

[76] Guzmán MG, Kourí G, Valdés L, Bravo J, Vázquez S, Halstead SB (2002). Enhanced severity of secondary dengue-2 infections: death rates in 1981 and 1997 Cuban outbreaks. Rev Panam Salud Publica.

[77] Yung C-F, Lee K-S, Thein T-L, Tan L-K, Gan VC, Wong JGX (2015). Dengue serotype-specific differences in clinical manifestation, laboratory parameters and risk of severe disease in adults, Singapore. Am J Trop Med Hyg.

[78] Vicente CR, Herbinger K-H, Fröschl G, Malta Romano C, de Souza Areias Cabidelle A, Cerutti Junior C (2016). Serotype influences on dengue severity: a cross-sectional study on 485 confirmed dengue cases in Vitória, Brazil. BMC Infect Dis.

[79] Wang W-K, Chao D-Y, Kao C-L, Wu H-C, Liu Y-C, Li C-M (2003). High levels of plasma dengue viral load during Defervescence in patients with dengue Hemorrhagic fever: implications for pathogenesis. Virology..

[80] Vaughn DW, Green S, Kalayanarooj S, Innis BL, Nimmannitya S, Suntayakorn S (2000). Dengue viremia titer, antibody response pattern, and virus serotype correlate with disease severity. J Infect Dis.

[81] Funk A, Truong K, Nagasaki T, Torres S, Floden N, Balmori Melian E (2010). RNA structures required for production of subgenomic flavivirus RNA. J Virol.

[82] Chapman EG, Costantino DA, Rabe JL, Moon SL, Wilusz J, Nix JC (2014). The structural basis of pathogenic subgenomic flavivirus RNA (sfRNA) production. Science..

[83] Roby JA, Pijlman GP, Wilusz J, Khromykh AA (2014). Noncoding subgenomic flavivirus RNA: multiple functions in West Nile virus pathogenesis and modulation of host responses. Viruses..

[84] Manokaran G, Finol E, Wang C, Gunaratne J, Bahl J, Ong EZ (2015). Dengue subgenomic RNA binds TRIM25 to inhibit interferon expression for epidemiological fitness. Science..

[85] Schnettler E, Sterken MG, Leung JY, Metz SW, Geertsema C, Goldbach RW (2012). Noncoding flavivirus RNA displays RNA interference suppressor activity in insect and mammalian cells. J Virol.

[86] Moon SL, Anderson JR, Kumagai Y, Wilusz CJ, Akira S, Khromykh AA (2012). A noncoding RNA produced by arthropod-borne flaviviruses inhibits the cellular exoribonuclease XRN1 and alters host mRNA stability. RNA.

[87] Chang R-Y, Hsu T-W, Chen Y-L, Liu S-F, Tsai Y-J, Lin Y-T (2013). Japanese encephalitis virus non-coding RNA inhibits activation of interferon by blocking nuclear translocation of interferon regulatory factor 3. Vet Microbiol.

[88] Green AM, Beatty PR, Hadjilaou A, Harris E (2014). Innate immunity to dengue virus infection and subversion of antiviral responses. J Mol Biol.

[89] Kao Y-T, Lai MMC, Yu C-Y (2018) How dengue virus circumvents innate immunity. Front Immunol [Internet]. [cited 2020 Sep 2];9. Available from: https://www.ncbi.nlm.nih.gov/pmc/articles/PMC6288372/10.3389/fimmu.2018.02860 PMC6288372 30564245

[90] Halstead SB, Lan NT, Myint TT, Shwe TN, Nisalak A, Kalyanarooj S (2002). Dengue hemorrhagic fever in infants: research opportunities ignored. Emerg Infect Dis.

[91] Hung NT, Lei H-Y, Lan NT, Lin Y-S, Huang K-J, Lien LB (2004). Dengue Hemorrhagic fever in infants: a study of clinical and cytokine profiles. J Infect Dis.

[92] Halstead SB (1970). Observations related to pathogensis of dengue hemorrhagic fever. VI. Hypotheses and discussion. Yale J Biol Med.

[93] Halstead SB (2015) Pathogenesis of dengue: dawn of a new era. F1000Research, 4 10.12688/f1000research.7024.1 PMC4754012 26918141

[94] Halstead SB, Chow JS, Marchette NJ (1973). Immunological enhancement of dengue virus replication. Nat New Biol.

[95] Halstead SB, Marchette NJ, Sung Chow JS, Lolekha S (1976). Dengue virus replication enhancement in peripheral blood leukocytes from immune human beings. Proc Soc Exp Biol Med.

[96] Halstead SB, O’Rourke EJ (1977). Dengue viruses and mononuclear phagocytes. I. Infection enhancement by non-neutralizing antibody. J Exp Med.

[97] Chaichana P, Okabayashi T, Puiprom O, Sasayama M, Sasaki T, Yamashita A (2014). Low levels of antibody-dependent enhancement in vitro using viruses and plasma from dengue patients. PLoS One.

[98] Goncalvez AP, Engle RE, St Claire M, Purcell RH, Lai C-J (2007). Monoclonal antibody-mediated enhancement of dengue virus infection in vitro and in vivo and strategies for prevention. Proc Natl Acad Sci U S A.

[99] Guzman MG, Alvarez M, Rodriguez-Roche R, Bernardo L, Montes T, Vazquez S (2007). Neutralizing antibodies after infection with dengue 1 virus. Emerg Infect Dis.

[100] Guzmán MG, Kouri G, Valdes L, Bravo J, Alvarez M, Vazques S (2000). Epidemiologic studies on Dengue in Santiago de Cuba, 1997. Am J Epidemiol.

[101] Takada A, Feldmann H, Ksiazek TG, Kawaoka Y (2003). Antibody-dependent enhancement of Ebola virus infection. J Virol.

[102] Chan-Hui P-Y, Swiderek KM (2016). Immunological considerations for developing antibody therapeutics for influenza A. Hum Vaccin Immunother.

[103] Katzelnick LC, Gresh L, Halloran ME, Mercado JC, Kuan G, Gordon A (2017). Antibody-dependent enhancement of severe dengue disease in humans. Science.

[104] Halstead SB, Mahalingam S, Marovich MA, Ubol S, Mosser DM (2010). Intrinsic antibody-dependent enhancement of microbial infection in macrophages: disease regulation by immune complexes. Lancet Infect Dis.

[105] Ubol S, Phuklia W, Kalayanarooj S, Modhiran N (2010). Mechanisms of immune evasion induced by a complex of dengue virus and preexisting enhancing antibodies. J Infect Dis.

[106] King CA, Anderson R, Marshall JS (2002). Dengue virus selectively induces human mast cell chemokine production. J Virol.

[107] Brown MG, Hermann LL, Issekutz AC, Marshall JS, Rowter D, Al-Afif A (2011). Dengue virus infection of mast cells triggers endothelial cell activation. J Virol.

[108] Sridhar S, Luedtke A, Langevin E, Zhu M, Bonaparte M, Machabert T (2018). Effect of dengue serostatus on dengue vaccine safety and efficacy. N Engl J Med.

[109] Hadinegoro SR, Arredondo-García JL, Capeding MR, Deseda C, Chotpitayasunondh T, Dietze R (2015). Efficacy and long-term safety of a dengue vaccine in regions of endemic disease. N Engl J Med.

[110] Martínez-Vega RA, Carrasquila G, Luna E, Ramos-Castañeda J (2017). ADE and dengue vaccination. Vaccine.

[111] Revilla F (2016) https://www.facebook.com/pahowho. PAHO/WHO | XXIV Technical Advisory Group (TAG) Meeting on Vaccine-preventable Diseases [Internet]. Pan American Health Organization/World Health Organization. [cited 2019 Mar 11]. Available from: https://www.paho.org/hq/index.php?option=com_content&view=article&id=12214:2016-xxiv-technical-advisory-group-tag-meeting&Itemid=40296&lang=en

[112] Ferguson NM, Rodríguez-Barraquer I, Dorigatti I, Mier-y-Teran-Romero L, Laydon DJ, Cummings DAT (2016). Benefits and risks of the Sanofi-Pasteur dengue vaccine: modeling optimal deployment. Science..

[113] Simmons CP, McPherson K, Van Vinh CN, Hoai Tam DT, Young P, Mackenzie J (2015). Recent advances in dengue pathogenesis and clinical management. Vaccine..

[114] Rivino L, Kumaran EAP, Jovanovic V, Nadua K, Teo EW, Pang SW (2013). Differential targeting of viral components by CD4+ versus CD8+ T lymphocytes in dengue virus infection. J Virol.

[115] Bozza FA, Cruz OG, Zagne SMO, Azeredo EL, Nogueira RMR, Assis EF (2008). Multiplex cytokine profile from dengue patients: MIP-1beta and IFN-gamma as predictive factors for severity. BMC Infect Dis.

[116] Kurane I, Matsutani T, Suzuki R, Takasaki T, Kalayanarooj S, Green S (2011). T-cell responses to dengue virus in humans. Trop Med Health..

[117] Green S, Pichyangkul S, Vaughn DW, Kalayanarooj S, Nimmannitya S, Nisalak A (1999). Early CD69 expression on peripheral blood lymphocytes from children with dengue hemorrhagic fever. J Infect Dis.

[118] Tsai T-T, Chuang Y-J, Lin Y-S, Wan S-W, Chen C-L, Lin C-F (2013). An emerging role for the anti-inflammatory cytokine interleukin-10 in dengue virus infection. J Biomed Sci.

[119] Pang T, Cardosa MJ, Guzman MG (2007). Of cascades and perfect storms: the immunopathogenesis of dengue haemorrhagic fever-dengue shock syndrome (DHF/DSS). Immunol Cell Biol.

[120] Agarwal R, Chaturvedi UC, Misra A, Mukerjee R, Kapoor S, Nagar R (1998). Production of cytotoxic factor by peripheral blood mononuclear cells (PBMC) in patients with dengue haemorrhagic fever. Clin Exp Immunol.

[121] Chaturvedi UC, Shrivastava R, Tripathi RK, Nagar R (2007). Denguevirus-specific suppressor T cells: current perspectives. FEMS Immunol Med Microbiol.

[122] Klenerman P, Zinkernagel RM (1998). Original antigenic sin impairs cytotoxic T lymphocyte responses to viruses bearing variant epitopes. Nature..

[123] Kinjyo I, Inoue H, Hamano S, Fukuyama S, Yoshimura T, Koga K (2006). Loss of SOCS3 in T helper cells resulted in reduced immune responses and hyperproduction of interleukin 10 and transforming growth factor-beta 1. J Exp Med.

[124] Tian Y, Grifoni A, Sette A, Weiskopf D (2019). Human T cell response to dengue virus infection. Front Immunol [Internet]. [cited 2020 Sep 3];10. Available from: https://www.frontiersin.org/articles/10.3389/fimmu.2019.02125/full 10.3389/fimmu.2019.02125 PMC6737489 31552052

[125] Malavige G, Fernando N, Ogg G (2011). Pathogenesis of dengue viral infections. Sri Lanka J Infect Dis.

[126] Lan NTP, Hirayama K (2011). Host genetic susceptibility to severe dengue infection. Trop Med Health.

[127] LaFleur C, Granados J, Vargas-Alarcon G, Ruíz-Morales J, Villarreal-Garza C, Higuera L (2002). HLA-DR antigen frequencies in Mexican patients with dengue virus infection: HLA-DR4 as a possible genetic resistance factor for dengue hemorrhagic fever. Hum Immunol.

[128] Green S, Vaughn DW, Kalayanarooj S, Nimmannitya S, Suntayakorn S, Nisalak A (1999). Early immune activation in acute dengue illness is related to development of plasma leakage and disease severity. J Infect Dis.

[129] Perez AB, Sierra B, Garcia G, Aguirre E, Babel N, Alvarez M (2010). Tumor necrosis factor-alpha, transforming growth factor-β1, and interleukin-10 gene polymorphisms: implication in protection or susceptibility to dengue hemorrhagic fever. Hum Immunol.

[130] García G, Sierra B, Pérez AB, Aguirre E, Rosado I, Gonzalez N (2010). Asymptomatic dengue infection in a Cuban population confirms the protective role of the RR variant of the FcgammaRIIa polymorphism. Am J Trop Med Hyg..

[131] Loke H, Bethell D, Phuong CXT, Day N, White N, Farrar J (2002). Susceptibility to dengue hemorrhagic fever in Vietnam: evidence of an association with variation in the vitamin d receptor and fc gamma receptor IIa genes. Am J Trop Med Hyg.

[132] Soundravally R, Hoti SL (2007). Immunopathogenesis of dengue hemorrhagic fever and shock syndrome: role of TAP and HPA gene polymorphism. Hum Immunol.

[133] Soo K-M, Khalid B, Ching S-M, Tham CL, Basir R, Chee H-Y (2017). Meta-analysis of biomarkers for severe dengue infections. PeerJ..

[134] Sehrawat P, Biswas A, Kumar P, Singla P, Wig N, Dar L, et al (2018) Role of cytokines as molecular marker of dengue Severity. Mediterr J Hematol Infect Dis [Internet]. [cited 2018 Sep 18];10(1). Available from: https://www.ncbi.nlm.nih.gov/pmc/articles/PMC5937971/10.4084/MJHID.2018.023 PMC5937971 29755701

[135] Tissera H, Rathore APS, Leong WY, Pike BL, Warkentien TE, Farouk FS (2017). Chymase level is a predictive biomarker of dengue hemorrhagic fever in pediatric and adult patients. J Infect Dis.

[136] Punyadee N, Mairiang D, Thiemmeca S, Komoltri C, Pan-ngum W, Chomanee N (2015). Microparticles provide a novel biomarker to predict severe clinical outcomes of dengue virus infection. J Virol.

[137] Ouyang X, Jiang X, Gu D, Zhang Y, Kong SK, Jiang C (2016). Dysregulated serum MiRNA profile and promising biomarkers in dengue-infected patients. Int J Med Sci.

[138] Cheng Z, Ma R, Tan W, Zhang L (2014). MiR-152 suppresses the proliferation and invasion of NSCLC cells by inhibiting FGF2. Exp Mol Med.

[139] Swaminathan G, Martin-Garcia J, Navas-Martin S (2013). RNA viruses and microRNAs: challenging discoveries for the 21st century. Physiol Genomics.

